# Reduced fecal short‐chain fatty acids in hispanic children with ulcerative colitis

**DOI:** 10.14814/phy2.14918

**Published:** 2021-07-18

**Authors:** Sarah Rotondo‐Trivette, Beibei Wang, Yihui Luan, Oliver Fiehn, Fengzhu Sun, Sonia Michail

**Affiliations:** ^1^ Keck School of Medicine University of Southern California Los Angeles California USA; ^2^ Molecular and Computational Biology Program Department of Biological Sciences University of Southern California Los Angeles California USA; ^3^ School of Mathematics Shandong University Jinan Shandong China; ^4^ Children’s Hospital of Los Angeles Los Angeles California USA

**Keywords:** pediatrics, short‐chain fatty acids, ulcerative colitis

## Abstract

**Background:**

It is known that patients with ulcerative colitis (UC) have reduced numbers of short‐chain fatty acid (SCFA) producing bacteria and reduced SCFA concentration in feces. There is also evidence that Hispanic patients have increased incidence of UC and increased likelihood of developing disease at a younger age. To understand why this might be, we compared fiber intake and fecal SCFA concentrations in Hispanic children with UC and non‐Hispanic children with UC.

**Methods:**

In this cross‐sectional study conducted at the Children's Hospital of Los Angeles, stool was collected from 22 Hispanic and 31 non‐Hispanic children with UC. SCFAs in the stool were quantified using mass spectrometry. Diet information was collected at the time of stool collection using food frequency questionnaires.

**Results:**

Acetic acid, butyric acid, isovaleric acid, and propionic acid concentrations are significantly lower in Hispanic children with UC compared to age, gender, and disease activity matched non‐Hispanic children with UC (*p* < 0.001). Butyric acid showed the most significant decrease (*p* = 1.6e‐7) There was no significant difference in fiber intake between Hispanic and non‐Hispanic children with UC.

**Conclusion:**

To our knowledge, this is the first study to find that Hispanic children with UC had further reduced SCFAs, independent of disease activity and fiber intake. It is possible that the reduction in SCFAs is related to the colonic disease in Hispanic patients with UC. This may provide more evidence to support the use of SCFA targeted therapies for UC.

## Graphical abstract

Hispanic children with ulcerative colitis have lower short chain fatty acids.

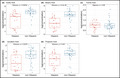

**Summary**

In this study, we demonstrate that Hispanic children with ulcerative colitis have reduced fecal short‐chain fatty acids compared to age and gender matched non‐Hispanic children with ulcerative colitis independent of disease activity and fiber intake.

## 1 INTRODUCTION

Ulcerative colitis (UC), a subtype of inflammatory bowel disease (IBD), is characterized by chronic inflammation of the colon. It is an important pediatric disease, with as many as 20%–25% of all cases presenting during childhood. The incidence of UC is constantly rising with alarming increases in pediatrics (Ye et al., 2020). Gut microbial dysbiosis has been suggested to play a role in the development in IBD (Goncalves et al., 2018). IBD patients have been shown to have dysbiosis with reduced numbers of short‐chain fatty acid (SCFA) producing bacteria (Goncalves 2018). SCFAs are defined as fatty acids having fewer than six carbons, including formic acid (C1), acetic acid (C2), propionic acid (C3), butyric acid (C4) and isovaleric acid(C5) (Holota et al., 2017). SCFAs are the main end‐products of anaerobic fermentation of dietary fiber by large intestine microbiota (Goncalves et al., 2018; Holota et al., 2017). They provide the major source of energy for colonocytes, exert anti‐inflammatory effects, and regulate the growth of known pathogens (Goncalves et al., 2018; Holota et al., 2017).

Acetic, propionic, and butyric acid account for more than 95% of SCFAs in the gut (Lavelle & Sokol, 2020; Sun et al., 2017; Tang et al., 2019). Butyric acid is particularly well studied and has been shown to promote intestinal barrier function and exert anti‐inflammatory effects on both intestinal epithelium and immune cells. Administration of SCFAs, especially butyrate, via enemas has shown positive results in reduction of human colitis severity (Goncalves et al., 2018; Scheppach, 1996; Sun et al., 2017). Additionally, in a small pilot study that we previously published (Nusbaum et al., 2018), we demonstrated that the microorganisms known to produce butyrate, such as *Roseburia*, were reduced in children with UC. Fecal transplant restored some of these depleted bacteria and increased fecal butyrate in children who respond favorably to this therapy.

Historically, IBD was considered a disease that predominately affected white populations and much of the IBD‐related research was being focused on Caucasian individuals. However, more research suggests that IBD affects other ethnic groups. IBD is now increasingly recognized in diverse ethnic populations and the rapidly shifting demographics in the United States necessitate a better understanding of how IBD may affect Hispanics, especially in vulnerable populations such as children. Although data on Hispanic children with UC is unfortunately very scarce, it suggests increased incidence and increased likelihood of developing disease at a younger age (Abramson et al., 2010; Hattar et al., 2012). There is also some evidence that Hispanic patients with UC are more likely to have pancolonic disease than left‐sided colitis and are more likely to undergo colectomy than white patients (Afzali & Cross, 2016; Nguyen et al., 2006). To evaluate the differences in SCFA profiles between Hispanic children with UC and non‐Hispanic children, we compared fecal SCFA concentrations between the two groups. Since SCFAs can be influenced by dietary fiber intake, we also evaluated the fiber intake of a subset of these children. We report a significant decrease in fecal SCFAs in Hispanic children with UC despite consuming similar amounts of soluble and insoluble fiber in their diets. This suggests that the etiology of SCFA depletion is independent of substrate availability.

## 2 METHODS

### 2.1 Recruitment

This study was conducted at the Children's Hospital of Los Angeles (CHLA) under IRB # 16–00050. The subjects were recruited by the investigator and study team in their regular GI clinic visit, while inpatient or through the department list. We compared fecal SCFAs of non‐Hispanic children with UC and Hispanic children with UC and fiber intake between the two groups.

Fecal samples and relevant clinical information were collected from 53 children with UC. Stool collected was immediately placed on ice and transferred to CHLA within 24 h. At CHLA, stool was stored at −80°C before being transported to University of California, Davis for processing. Additionally, we collected diet information from 16 Hispanic children and 12 non‐Hispanic children with UC at the time of stool collection using food frequency questionnaires (Block & Subar, 1992; Block et al., 1992) to assess soluble and insoluble fiber intake.

### 2.2 Quantification of short‐chain fatty acids

SCFAs were quantified using a method we have previously described^8^. Briefly, 10 mg samples of feces were used for SCFA analysis at West Coast Metabolomics Center at the University of California, Davis. Metabolites were extracted with 700 μl of water, hydrochloric acid, and methyl *tert*‐butyl ether (5:1:1). Samples were homogenized using a Genogrinder at 1500 rpm for 30 s, shaken for 30 min at room temperature, and centrifuged for 2 min at 14000 rcf. A quantity of 500 μl of supernatant was transferred to a new tube and 0.1 g of anhydrous sodium sulfate was added to the supernatant for dehydration. A quantity of 25 μl of *N*‐*tert*‐butyldimethylsilyl‐*N*‐methyltrifluoroacetamide (MTBSTFA, Sigma‐Aldrich) was used for *tert*‐butyldimethylsilylation. Samples were then shaken at 80°C for 30 min and ready for injection. An Agilent 5977A GC‐quadrupole mass spectrometer was used for data acquisition in selected ion monitoring (SIM) mode. Raw data were processed using Agilent Mass Hunter Quantitative Analysis software (B.07.00) and data were quantified against authentic standards.

### 2.3 Statistical analysis

We first used logarithm to transform the raw concentration data. Q‐Q plots of log‐transformed concentrations of the SCFAs were used to see whether the data followed a normal distribution. If a SCFAs concentration was normally distributed in a population, the Q‐Q plot would be approximately linear. Shapiro–Wilk tests were also used to test whether the data was normally distributed. If the p value of the Shapiro–Wilk test was greater than 0.05, we could not reject the normal distribution of the data. As the data was found to be normally distributed, Student's *t* tests were performed to compare the differences of the SCFAs between UC patients with different ethnicities: Hispanic and non‐Hispanic.

Next, we compared the intake of soluble and insoluble fiber in both groups. Again, Q–Q plots of the data were used to see whether the data followed a normal distribution, as well as Shapiro–Wilk tests. Student's *t* tests were performed to see the differences between Hispanics and non‐Hispanics, as the data were normally distributed.

## 3 RESULTS

### 3.1 Demographics

As shown in Table 1, Hispanic and non‐Hispanic children included in this study were age and gender matched and had similar pediatric ulcerative colitis activity index (PUCAI) scores. Hispanic children had a higher rate of pancolitis, hospitalization, and need for blood transfusion. Non‐Hispanic children were mostly Caucasian (*n* = 25), followed by Asian (*n* = 3) and African American (*n* = 3).

**TABLE 1:** Demographics

	Hispanic	Non‐Hispanic	*p*‐values
Number	22	31	
Age in years (average)	16.82	16.13	0.46
Gender (% females)	45.45	45.16	0.98
PUCAI	41.14	43.71	0.57
Pancolitis	90.91%	74.19%	0.11
Hospitalization	68.18%	51.61%	0.23
Need for blood transfusion	40.91%	19.35%	0.10

### 3.2 Fecal short‐chain fatty acids

**FIGURE 1:**
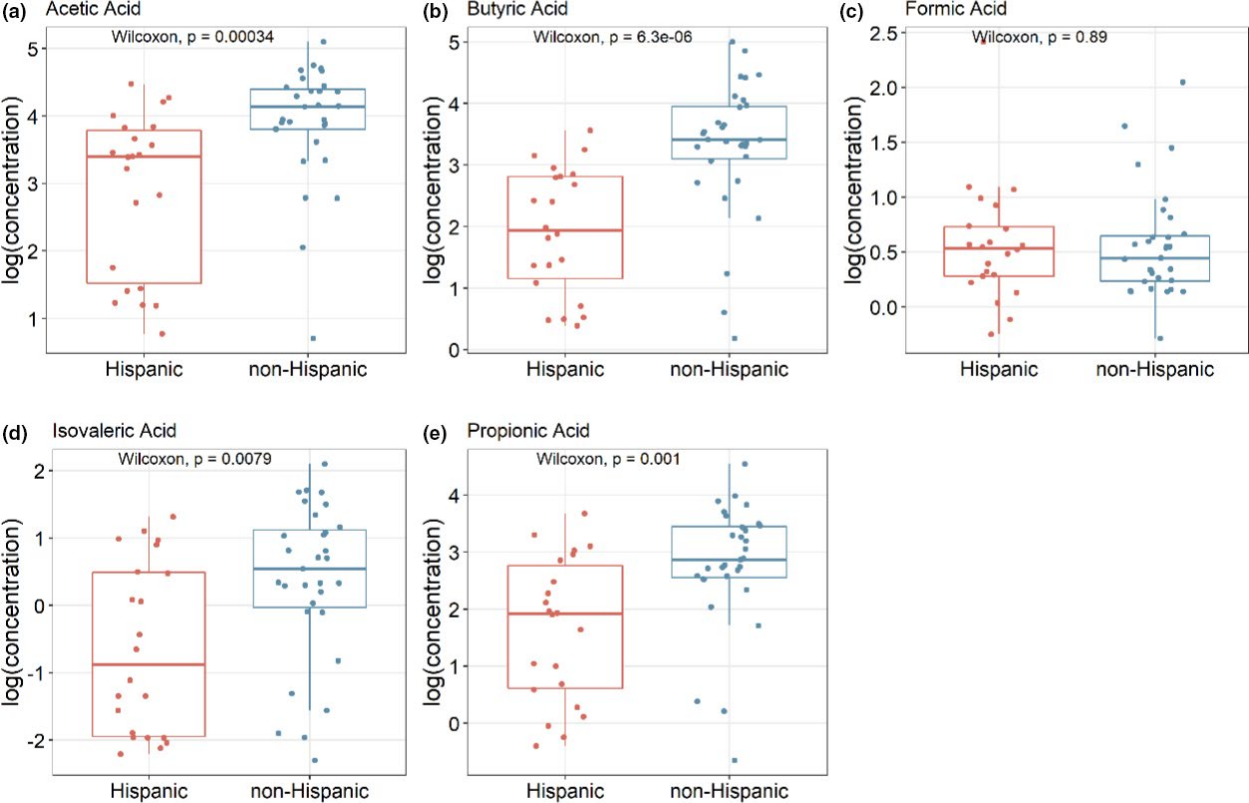
Box plot of the log transformed SCFA concentration (micrograms/milligram) for Hispanic UC (red) and non‐Hispanic UC (blue) samples. *p* values obtained using Wilcoxon tests

The raw SCFA concentrations are shown in Table 2. It is apparent that while there was a wide range of concentrations, acetic, butyric, and propionic acid concentrations were much higher than that of isovaleric and formic acid on average. As shown in Figure 1, acetic acid, butyric acid, isovaleric acid, and propionic acid were significantly decreased in Hispanic compared to non‐Hispanic (*p* < 0.001). Butyric acid, which is the best studied SCFA for its anti‐inflammatory effects, had the most significant difference between the two groups (*p* = 1.6e‐7). Formic acid levels in the two populations were similar (*p* > 0.05).

**TABLE 2:** Profiles of short‐chain fatty acid raw data (micrograms/milligram)

	Maximum	Minimum	Mean	Standard Deviation
Acetic Acid	164.06	2.02	49.52	35.60
Butyric Acid	148.42	1.20	28.32	30.79
Formic Acid	11.18	0.75	2.08	1.73
Isovaleric Acid	8.15	0.10	1.79	1.77
Propionic Acid	94.00	0.52	17.95	17.36

**FIGURE 2:**
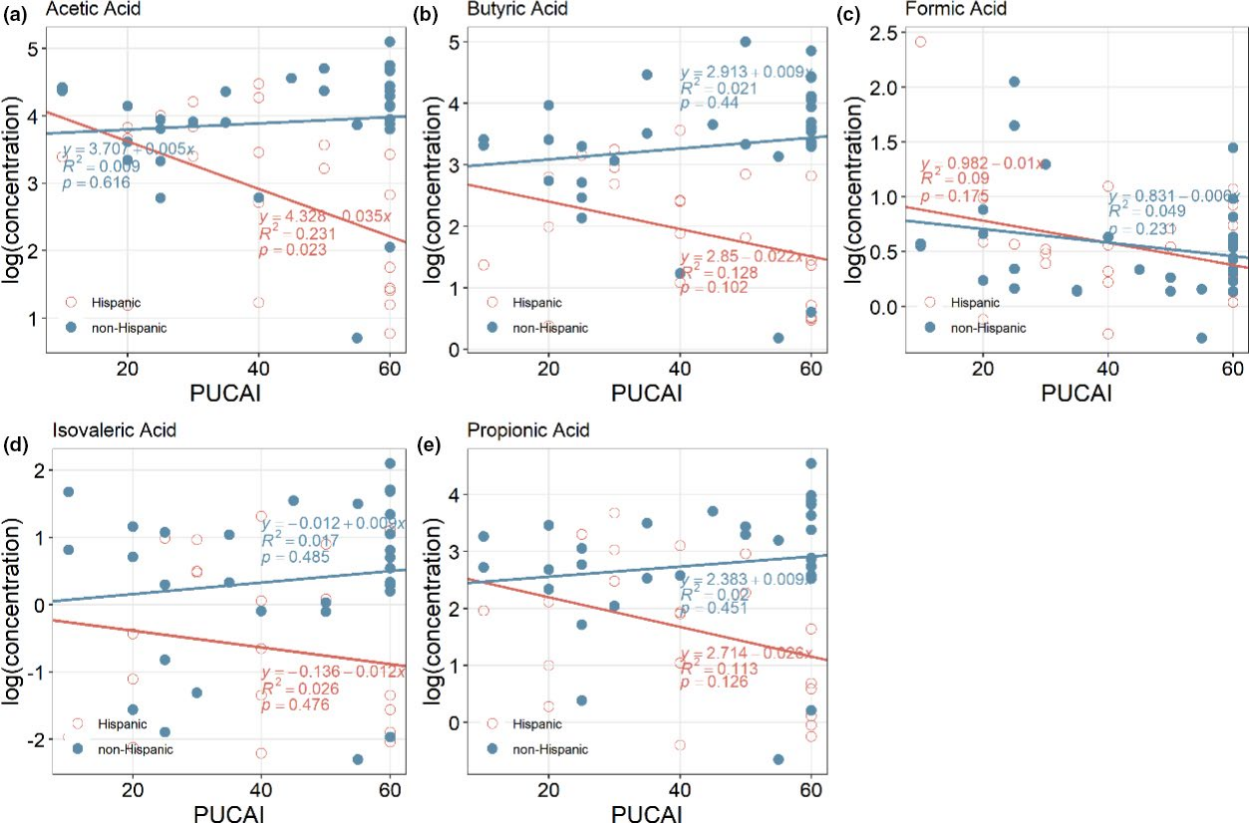
Scatter plot of the log transformed abundance changes for Acetic Acid (a), Butyric Acid (b), Formic Acid (c), Isovaleric Acid (d), and Propionic Acid (e) along with PUCAI for Hispanic (red) and non‐Hispanic (blue) UC patients. Linear fitted curve, the Goodness of Fit, as well as the p‐value from F test are shown in the figure

We then compared the abundance of the individual SCFAs relative to the PUCAI scores and as shown in Figure 2, Hispanic children with UC had an inverse relationship of fecal acetic acid and PUCAI scores.

### 3.3 Fiber Intake

**FIGURE 3:**
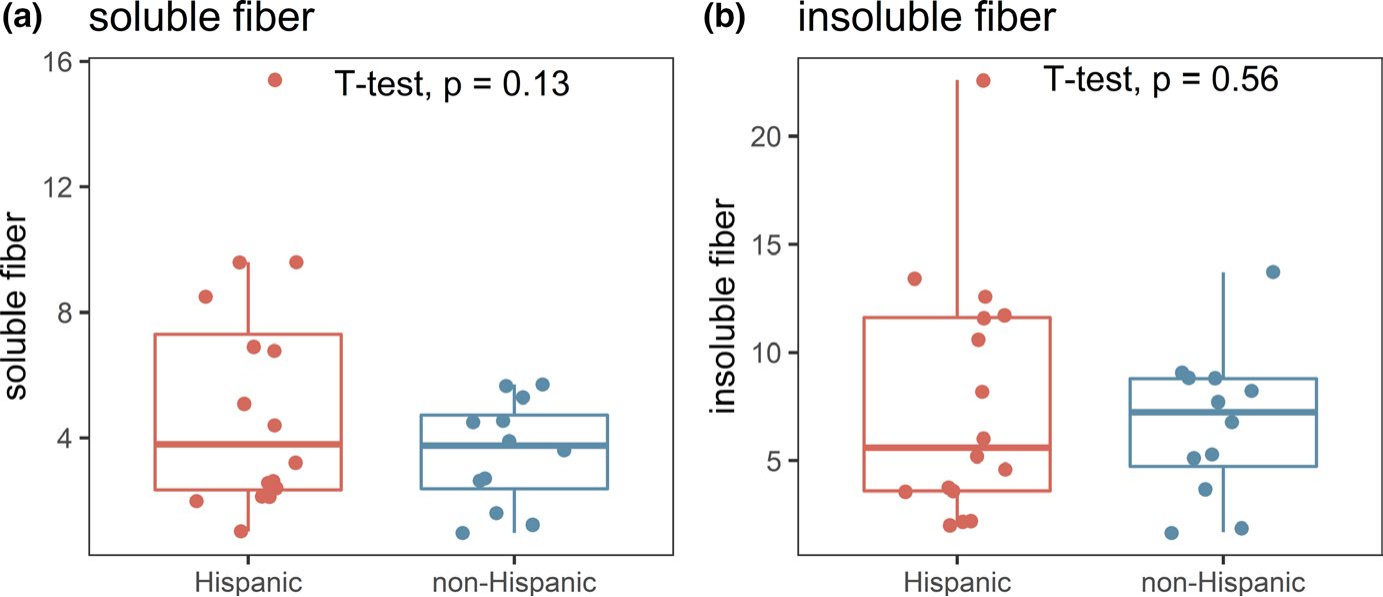
Intake of soluble and insoluble fiber in Hispanic and non‐Hispanic children with UC. P values obtained by t tests are also included

SCFAs are a product of bacterial fermentation of fiber in the gut. Therefore, decreased fiber intake would result in decreased SCFAs due to having less substrate available for fermentation. As seen in Figure 3, there was no significant difference in the soluble or insoluble fiber intake between this subset of Hispanic and non‐Hispanic children with UC (*p* > 0.05).

## 4 CONCLUSIONS

SCFAs are among the most abundant and best‐studied microbial metabolites. They are derived from commensal bacterial fermentation of indigestible dietary fibers in intestines and found in high concentrations in the healthy gut (Chen et al., 2019; Goncalves et al., 2018; Lavelle & Sokol, 2020; Tang et al., 2019). SCFAs influence immune system homeostasis and are a main energy source for colonocytes (Goncalves et al., 2018; Lavelle & Sokol, 2020; Tang et al., 2019). IBD patients have been previously shown to have reduced numbers of SCFA producing bacteria and reduced SCFA concentration in feces (Lavelle & Sokol, 2020; Sun et al., 2017; Tang et al., 2019; Treem et al., 1994). We found that Hispanic children with UC had further reduced fecal SCFAs compared to age and gender matched non‐Hispanic children with UC with similar disease severity.

A previous study found that SCFA concentrations correlated with UC disease severity scores (Treem et al., 1994). However, it was unclear if decreased SCFA concentration was a cause or effect of greater disease severity. Many patients in an acute flare of UC will restrict their fiber intake, which could confound this result by decreasing the fiber available to intestinal microbes for fermentation. In our study, PUCAI scores were similar in both Hispanic and non‐Hispanic children to allow a fair overall comparison. It is interesting that we discovered Hispanic children with UC had lower fecal concentrations of acetic, butyric, isovaleric, and propionic acids when they had higher PUCAI scores, more severe disease activity, and had higher fecal levels of these SCFAs with lower PUCAI scores, more mild disease. (Figure 2). This appears to be specific to the Hispanic children and was not seen in the non‐Hispanic cohort.

Disease activity or PUCAI scores refer to a cross‐sectional, moment‐in‐time assessment of inflammation, whereas disease severity includes more longitudinal and historical factors such as pancolitis, history of hospitalization, and need for blood transfusions (Pabla & Schwartz, 2020; Siegel et al., 2018). While our Hispanic cohort was selected in a manner that would match the non‐Hispanic cohort with regard to disease activity, our Hispanic children appeared to have more severe disease on account of having higher rates of pancolitis, hospitalizations, and history of blood transfusions.

In our pediatric population, we also sought to investigate whether the change in fecal SCFAs was related to decreased fiber intake in Hispanic populations. The role of the diet in Hispanic populations is an important factor to consider, as diet is deeply interconnected with culture and tradition in many communities and can impact the gut microbiome and SCFA production. A study by Damas et al showed that the majority of Hispanic patients with IBD changed their diet upon immigration to the USA (Damas et al., 2018). The authors suggested future studies to examine gene‐diet interactions to better understand underlying causes of IBD in Hispanics. However, results from the food frequency questionnaire (Block & Subar, 1992; Block et al., 1992) obtained from Hispanic and non‐Hispanic children at time of fecal sample collection, surprisingly did not demonstrate a decrease in soluble or insoluble fiber intake by Hispanic children (Figure 2). This suggests that the decrease in SCFAs may be related to lower production by the gut microbiota rather than decreased substrate in the diet. Even though the food frequency questionnaire was not obtained from all subjects, the subjects with diet history were a good representation of each population and PUCAI scores from subjects with dietary information were similar to those without dietary information.

To our knowledge, this is the first study to find that Hispanic children with UC had further reduced levels of SCFAs. Depleted fecal SCFAs do not appear to be related to decreased intake of fiber but is likely related to non‐substrate production processes, perhaps through the presence of fewer gut microbial SCFA producers. We previously demonstrated that fecal transplant could restore SCFA producers in UC patients (Nusbaum et al., 2018). However, while fecal transplant may be promising in changing the gut microbiome in pediatric UC, it has recently been associated with increased risk of serious infections (Michail et al., 2020). More targeted therapies for increasing SCFAs, such as butyrate supplementation, delivery of targeted butyrate producing organisms, or through targeted molecular pathway therapies related to the host genome–gut microbial interactions could confer the benefits of fecal transplant while minimizing the risk of infection.

To summarize, Hispanic children with UC have reduced fecal SCFA concentrations compared to age, gender matched non‐Hispanic children with similar overall disease activity but inversely correlates with individual PUCAI scores in Hispanic children. This reduction in SCFAs appears to be independent of fiber intake, both soluble and insoluble. It is possible that the more significant reduction in SCFAs is related to the disease presentation of Hispanic UC patients. Future studies will be important to further delineate the role of SCFAs, especially butyric acid, in maintaining colonic health in this population and the possibility of butyrate‐based targeted therapies.

## CONFLICT OF INTEREST

Sarah Rotondo‐Trivette has no conflicts of interest to disclose. Beibei Wang has no conflicts of interest to disclose. Yihui Luan has no conflicts of interest to disclose. Fengzhu Sun has no conflicts of interest to disclose. Sonia Michail has no conflicts of interest to disclose.

## AUTHOR CONTRIBUTIONS

Sarah Rotondo‐Trivette: Collected data, performed initial analysis, wrote the manuscript under guidance of Drs. Michail and Sun. Beibei Wang: Performed all the data analysis and interpretation under the direction of Drs. Luan and Sun. Yihui Luan: Supervised data analysis and interpretation. Oliver Fiehn: Assisted with data interpretation. Fengzhu Sun: Supervised data analysis and contributed to the development of the concept. Sonia Michail: planned the project, supervised sample collection and processing as well as supervised data management and writing manuscript.
